# Wireless Pulmonary Artery Pressure Monitor Implantation in a Patient with Duchenne Muscular Dystrophy

**DOI:** 10.1007/s00246-024-03459-z

**Published:** 2024-03-19

**Authors:** T. Miller Sisson, Juli Sublett-Smith, Elizabeth Dupont, Russel Hirsch, Angela Lorts, Chet Villa

**Affiliations:** 1 Department of Pediatrics, Cincinnati Children’s Hospital Medical Center, University of Cincinnati College of Medicine, Cincinnati, OH 45229 USA; 2 The Heart Institute, Cincinnati Children’s Hospital Medical Center, University of Cincinnati College of Medicine, Cincinnati, OH 45229 USA; 3 Cincinnati Children’s Hospital Medical Center, MLC 2003, 3333 Burnet Avenue, Cincinnati, OH 45229 USA

**Keywords:** Heart failure, Cardiomyopathy, Remote patient monitoring

## Abstract

Assessing heart failure progression in patients with Duchenne Muscular Dystrophy (DMD) is challenging given the multi-system nature of disease. Herein we describe the first case use of an implantable pulmonary artery pressure monitor and describe the potential clinical utility of this approach in patients with DMD.

## Case Report

The patient is a 23-year-old male with DMD who had been managed on chronic oral steroids since age 3. Of note, he lived remote from any major medical center, and owing to logistic difficulties, was only able to attend clinic periodically. Due to significant progressive weakness, he had been managed at home with sip ventilator during the day and bi-level ventilation at night. His most recent forced vital capacity from 7 years prior was 0.9 L (22% predicted). At presentation, he was on carvedilol 9.375 mg twice daily, lisinopril 2.5 mg daily, and spironolactone 25 mg daily. He had previously tolerated doses as high as carvedilol 12.5 mg twice daily and lisinopril 10 mg daily, but those doses had been sequentially decreased due to symptomatic hypotension since an admission for pneumonia 5 years prior to his current presentation.

On presentation to clinic, he complained of acute on chronic (> 3 years) fatigue and lower extremity pitting edema. Over the prior 6 months he described progressive cough and orthopnea. He also noted swelling and intermittent orthopnea since the previously noted admission for pneumonia.

His clinical exam was remarkable for a pulse of 84 beats/min, blood pressure 124/86 mmHg, oxygen saturations of 97% in room air, a weight of 103.9 kg and a BMI of 41 kg/m^2^. Jugular venous distention and hepatomegaly could not be assessed due to his body habitus/body position. Mild pitting edema was noted to the calf bilaterally, as well as non-pitting edema of the arms to the level of the bicep. There was no peripheral hyperpigmentation or ulceration noted.

His nt-BNP was 150 pg/mL, similar to the year prior (128 pg/mL) and slightly elevated from a measurement 5 years prior (109 pg/mL). Additional laboratory evaluation including hepatic and renal function were unchanged. Cystatin C GFR was stable over the previous 5 years (85–92 mL/min/1.73 m^2^) and the total bilirubin was normal. His cardiac MRI demonstrated a LVEF of 39% and RVEF of 51% in the absence of ventricular dilation (left ventricular end diastolic volume 68 mL/m^2^, right ventricular end diastolic volume 55 mL/m^2^). These findings were stable when compared with serial cardiac MRIs over the previous 3 years.

**Fig. 1:**
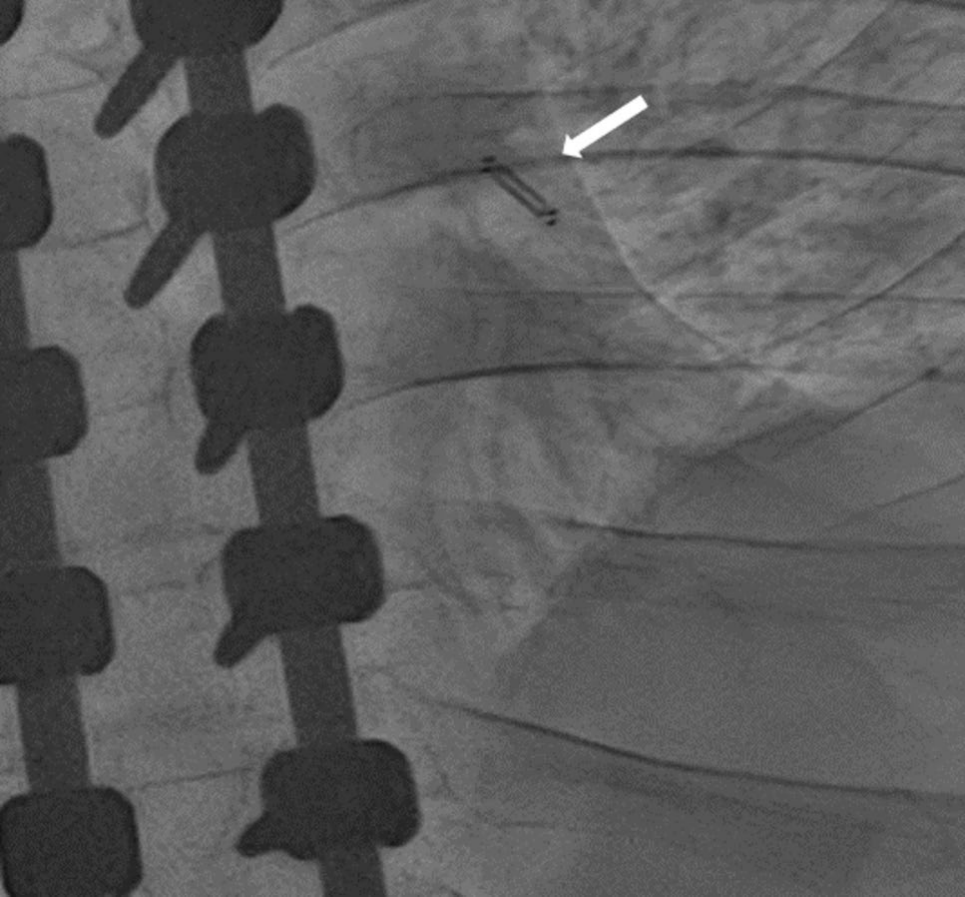
Wireless pulmonary artery pressure device (white arrow) in situ after completion of the procedure

He was initially managed with changes in salt and fluid intake as well as clinically driven diuretic management. However, due to persistence of symptoms and the challenges in differentiating heart failure and respiratory/swallow symptoms, the patient underwent right heart catheterization under minimal sedation. Hemodynamics revealed a low thermodilution cardiac index of 2.34 L/min/m^2^, and elevated right and left ventricular filling pressures [mean right atrial pressure 15 mmHg, mean pulmonary artery wedge pressure 27 mmHg, and a normal pulmonary vascular resistance (2.5 Wu m^2^)]. During the cardiac catheterization, and after a pulmonary angiogram demonstrated an appropriately sized left lower pulmonary artery (greater than 7 mm in diameter), a wireless pulmonary artery pressure device (CardioMEMS, Abbott Laboratories, Atlanta, GA) was implanted and calibrated successfully (Fig. 1).

The patient had an uneventful recovery from the catheterization and was discharged home on increased diuretic therapy. During the 24 months of follow-up, the patient has been able to transmit pulmonary artery recordings at regular intervals (ultimately spaced out to every 2–4 weeks once he was stable). Briefly, changes in this patient’s symptoms, such as worsening cough or dyspnea were compared to pulmonary artery pressure recordings, and in cases of increased pressure and symptomatic worsening, diuretic dosing was increased. Titrations such as these were managed remotely without requiring patient travel. The symptoms of cough and dyspnea have now completely resolved, and he has not required any further admissions.

## Discussion

This case represents the first known implantation of a wireless pulmonary artery pressure monitor in a patient with DMD. The use of this technology has not previously been reported in this patient population, and this case report demonstrates the potential utility of remote invasive monitoring given the challenges in assessing heart failure symptoms and medication management in non-ambulatory DMD patients with advanced multi-system disease.

In DMD, symptom assessment and medication titration may be challenging due to the nature of this multi-system disease. Furthermore, typical biomarkers of disease progression in DMD may be hard to interpret, and invasive hemodynamic assessment may be required to discern the contribution of heart failure to disease symptoms.

Improvement in respiratory and neuromuscular care have extended survival in DMD by delaying the onset of respiratory failure [1]. These improvements have unmasked the progression of cardiomyopathy which typically begins in the teenage years and progresses with age [2]. By the time cardiomyopathy progresses, patients often are non-ambulatory and have appreciable respiratory weakness. The challenges in documenting disease progression and attributing symptom progression to cardiac disease are further complicated by the limitations of physical examination, and laboratory and radiographic testing in patients with advanced disease. Furthermore, obesity commonly limits clinical assessment of jugular venous distention, while lung volume loss and scoliosis/patient positioning can make assessment of hepatomegaly a challenge. Lower extremity edema is common in DMD, especially in patients with long standing loss of ambulation and minimal residual lower extremity strength [3]. Additionally, there are no comprehensive studies describing the progression of laboratory biomarkers of disease progression such as natriuretic peptides. Existing case series suggest modified “normal values” may be needed in DMD, as appreciable rises may be a late finding [4]. Similarly, in our case, persistently low nt-BNP values (100–150 pg/mL) and “stably” depressed left ventricular systolic dysfunction (LVEF 40% ± 2%) had provided false reassurance that his symptoms were driven by respiratory progression rather than heart. In his case, cardiac catheterization was immensely useful in understanding the cardiac contribution to his overall disease process.

The implantation of the remote monitoring device allowed for successful outpatient management. Considering his course since implantation, with diuretic adjustment, and with no further admissions for pneumonia or respiratory disease (with no changes in pulmonary management), we believe this patient had been subject to undiagnosed pulmonary venous congestion secondary to left atrial hypertension that contributed to his respiratory symptoms and pulmonary admissions for many years. His case is consistent with published data indicating that remote patient monitoring using pulmonary artery pressure monitoring is associated with lower heart failure hospitalizations and heart failure related costs in non-DMD patients [5–7].

This case, as well as the evolving clinical experience suggests that centers should consider cardiac catheterization with potential use of implantable pressure monitors where applicable to help discern the potential cardiac contribution to respiratory symptoms. We would make the further argument that this should be done earlier in the course of the disease when the risk of the initial catheterization would be substantially lower. It is also worth noting, that contractures and/or appreciable scoliosis would be potential concerns in this population. Fortunately, this patient had no contractures impacting the ability to access the vasculature, and had his scoliosis addressed previously.

Finally, while there is ongoing debate regarding the impact of remote patient monitoring in specific populations, this case demonstrates its potential use in a rare disease cohort that may have limited alternative options. This approach is also finding potential application in children with congenital heart disease and heart failure [8].

## Conclusion

This patient’s case suggests implantable pulmonary artery pressure guided management can assist clinical management for DMD patients. The potential utility of this approach is especially important since many adults with DMD have limited local access to adult care providers with experience in DMD related heart failure, and long-distance management is thus attainable. Further study of this innovative approach is warranted, and collective experience to understand the overall impact is vitally important.

## Abbreviations

DMD: Duchenne muscular dystrophy
GFR: Glomerular filtration rate
kg: Kilogram
LVEF: Left ventricular ejection fraction
L: Liter
m: Meter
mg: Milligram
min: Minute
mL: Milliliter
mm: Millimeter
mmHg: Millimeters of mercury
MRI: Magnetic resonance imaging
nt-BNP: Aminoterminal B-type natriuretic peptide
PA: Pulmonary artery
pg: Picogram
RVEF: Right ventricular ejection fraction
Wu: Wood unit
